# Nanoparticles: A Challenging Vehicle for Neural Stimulation

**DOI:** 10.3389/fnins.2016.00105

**Published:** 2016-03-23

**Authors:** Elisabetta Colombo, Paul Feyen, Maria Rosa Antognazza, Guglielmo Lanzani, Fabio Benfenati

**Affiliations:** ^1^Center for Synaptic Neuroscience and Technology, Istituto Italiano di TecnologiaGenova, Italy; ^2^Center for Nano Science and Technology, Istituto Italiano di TecnologiaMilan, Italy; ^3^Department of Physics, Politecnico di MilanoMilan, Italy; ^4^Department of Experimental Medicine, Università di GenovaGenova, Italy

**Keywords:** nanoparticle, neural activity modulation, neurostimulation, neural prosthetics, nanobeads

## Abstract

Neurostimulation represents a powerful and well-established tool for the treatment of several diseases affecting the central nervous system. Although, effective in reducing the symptoms or the progression of brain disorders, the poor accessibility of the deepest areas of the brain currently hampers the possibility of a more specific and controlled therapeutic stimulation, depending on invasive surgical approaches and long-term stability, and biocompatibility issues. The massive research of the last decades on nanomaterials and nanoscale devices favored the development of new tools to address the limitations of the available neurostimulation approaches. This mini-review focuses on the employment of nanoparticles for the modulation of the electrophysiological activity of neuronal networks and the related transduction mechanisms underlying the nanostructure-neuron interfaces.

## Introduction

In 1939 it was reported that “*Nervous messages are invariably associated with an electrical change known as the action potential*,” representing day-one of electrophysiology and the dawning of neural stimulation (Hodgkin and Hukley, 1939). Nearly 40 years later, cochlear implants for instance were commercially available, successfully providing functional hearing to thousands of deafened people. Along the development of conventional electrical stimulation techniques, less invasive methodologies such as transcranial magnetic or ultrasonic stimulation have been employed for motor rehabilitation or therapy of neuropsychiatric disorders (Dayan et al., 2013; Legon et al., 2014; Noda et al., 2015). A very recent advancement for the modulation of neuronal activity is the use of light stimulation (Antognazza et al., 2014). Illumination of neurons can affect their activity either by light-induced temperature raise, exploiting the intrinsic physical, and chemical dynamics of neuronal membranes (Duke et al., 2013; Farah et al., 2013; Martino et al., 2015), or by photovoltaic interfaces that transduce light into an electrical stimulation (Ghezzi et al., 2011, 2013; Mandel et al., 2013). In addition, optogenetics has shown an innovative potential to interrogate neural circuits with unprecedented precision and specificity (Packer et al., 2013) and treat several diseases such as hereditary blindness, epilepsy, or Parkinson's disease (Busskamp and Roska, 2011) with the possibility of wireless implants for chronic light stimulation (Montgomery et al., 2015).

Considering the overall historical timeline, neural stimulation has developed at the pace of silicon technology and its progressively cutting-edge miniaturization. The coming out of the so-called Michigan and later Utah microelectrode arrays demonstrated in fact that neuroscience theranostics could take advantage of implantable neural probes able to target multiple brain areas with high spatial resolution (Wise et al., 1970; Jones et al., 1992). The downscaling and development of stimulation electrodes and interfaces is still an actual challenge for the scientific community, recently encouraged by the success obtained in clinical cases of vision restoration (Ho A. C. et al., 2015; Stingl et al., 2015) or deep brain stimulation (DBS) treatment of neural disorders (McIntyre et al., 2015; Schoenberg et al., 2015).

In the last decade, the scientific community described nanoscale neural interfaces as a valuable tool for drug delivery or monitoring of neuronal activity (Kotov et al., 2009; Luan et al., 2014). State of the art nanoelectrodes were successfully employed to localize epileptic foci in mice (Kang et al., 2014), while plasmonic nanoantennas combined the monitoring of extracellular activity with enhanced chemical analysis of neuronal cultures (Dipalo et al., 2015). Nonetheless, nanoelectrodes technology is not yet mature enough to provide a reliable stimulation or to flawlessly couple biocompatibility and neurointerfacing issues to the edge of fabrication techniques.

Freestanding nanoparticles (NPs) face instead moderate problems concerning processing procedures, relying on established fabrication techniques (e.g., chemical, colloidal synthesis), or more inorganic-oriented techniques (epitaxial growth or vapor deposition). Notwithstanding the preservation of a nanoscale dimension together with an easily functionalized surface, NPs raise concerns about their toxicity. Although, many aspects need further investigation, it has been demonstrated that the physicochemical properties like size and shape, surface charge or composition play a crucial role. Moreover, NPs lead frequently to the generation of reactive oxygen species that cause noxious secondary effects like DNA damage, inhibition of cell growth, and mitochondrial functional loss, all eventually promoting cell death (Kang et al., 2015). Drug delivery has also been extensively pursued with NPs of a wide variety of compositions, from silica to graphene oxide (Hu et al., 2015; Xiao et al., 2015) and from liposomes to biodegradable polymers (Wei et al., 2015; Zou et al., 2015). Another extensive use of NPs deals with hyperthermal therapies for cancer treatment that mostly exploits the magnetic susceptibility of metal NPs (Hauser et al., 2015). Recently, NPs embedded into conductive polymers have been reported to act as topographic guidance for neurons (Ho D. et al., 2015), while carbon-based materials among other nanostructures (Polak and Shefi, 2015) have been shown to be an excellent tool to drive nerve tissue repair and cell differentiation (Fabbro et al., 2013).

The landscape of NPs applications in neural interfaces is vast and beyond the scope of this report, but undeniably showing the focus that lately research attributed to nanomedicine. Nanotechnology and neuroscience are still struggling to find a compromise between toxicity, functionality, and access to the brain from the periphery for the methodologies based on NPs. Overcoming the physical constraints of other stimulation techniques, such as invasive surgical implants or genetic modifications, would be a major breakthrough in therapeutic stimulation.

Given these premises, we will focus this mini-review on the main NPs-based techniques employed so far to induce changes in neuronal activity and on the origin of the phenomena responsible for neural interfacing with these nanomaterials.

## Thermal stimulation

Temperature variations induce a perturbation of neuronal activity originating either from the intrinsic properties of the plasma membrane or from the temperature sensitivity of membrane proteins. Spatially localized temperature gradients on neuronal tissues were reported to be responsible for the increase in membrane capacitance and the consequent trigger of action potential firing (Shapiro et al., 2012; Duke et al., 2013). On the contrary, slow and prolonged heating is known to inhibit neuronal activity due to a predominant contribution of ionic channel modulation (Duke et al., 2013). In addition, the specific temperature sensitivity of the transient receptor potential vanilloid (TRPV) (Caterina et al., 1997) constitutes another extensively studied trigger of neuronal activity.

Gold nanoparticles (AuNPs) and nanorods (AuNRs) exposed to visible or near infrared (NIR) stimuli express surface plasmon resonance that is partly converted into thermal dissipation. This phenomenon has recently been reported to mediate highly localized heat-induced changes in neuronal membrane capacitance (Paviolo and Stoddart, 2015). The thermal transduction mediated by AuNPs and AuNRs proved to be a versatile tool to affect neuronal activity, triggering membrane depolarization from 0.025 to 25 ms illumination pulses (Yong et al., 2014) and action potential firing up to 200 Hz stimulation frequencies (Carvalho-de-Souza et al., 2015), given a surface functionalization to minimize thermal damage and aggregation. Neuronal excitation has been similarly reported using AuNRs to activate temperature-sensitive channels in the rat sciatic nerve *in vivo* upon NIR illumination, where a temperature increase of 6°C resulted in 5.7 times higher neuronal responsivity (Eom et al., 2014). Photo-Absorber Induced Neural-Thermal Stimulation (PAINTS) has provided another interesting way to excite neurons, exploiting the sub-millisecond thermal transients experienced by cells around micrometric fluorescent dyes upon optical stimulation (Farah et al., 2013).

Interestingly, PEGylated AuNRs were also demonstrated to effectively drive thermal inhibition of the spiking activity of cultured neurons exposed to prolonged 785 nm laser pulses (Yoo et al., 2014). NRs bound to neuronal membranes contributed significantly more to the photothermal silencing than the ones suspended in the medium prior to binding, demonstrating once more the high potential for targeted stimulation. Although, the mechanisms underlying the photothermal stimulation need further investigations, an involvement of thermo-sensitive K^+^ channel TREK-1 is suggested to be implicated.

A similar feature was reported by using thin films of photovoltaic semiconductor polymers, which exerted both depolarizing and hyperpolarizing effects on cell lines and neurons upon illumination as a function of the illumination time (Ghezzi et al., 2013; Martino et al., 2015; Feyen et al., 2016). We think these reports provided the foundation for a downscaling of photovoltaic polymeric films to *in vivo* injectable NPs for neural stimulation.

Neuronal stimulation has also been achieved by inducing a localized temperature raise through the heating of NPs subjected to a magnetic field. Magnetic stimulation in the macro-scale is in fact a common tool in neurophysiology thanks to the possibility to reach deep areas of the brain tissue. Manganese ferrite NPs have been used to generate a behavioral response in live worms upon magnetic-induced temperature rise of 14°C (Huang et al., 2010). Given the prolonged duration of the stimulation and the evoked temperature increase, it is believed that the activation of TRPV1 channels by the magnetothermal stimulation represent the major mechanism involved in this effect. Indeed, in a recent work, Chen et al. (2015) showed the co-localization of transfected TRPV1 channels and neuronal activation upon magnetic field application in mice injected with iron oxide NPs by *in vivo* imaging of c-Fos, an immediate early gene that is upregulated by neuronal activation.

Magnetic fields compete with ultrasounds (US) as non-invasive and low toxicity sources for therapeutic tissue stimulation. A novel thermal stimulation which is also worth mentioning has in fact exploited nano-piezoelectric transducers to modulate Ca^2+^ channels activity in SH-SY5Y neuroblastoma cells upon 1 MHz US stimulation (Marino et al., 2015).

## Electric and electromagnetic stimulation

Energy transduction pathways of high interest for neurostimulation are those resulting in changes in neural tissue's local electric fields. Traditionally, such approaches have been carried out using metal electrodes in contact with cells to achieve a capacitive coupling or by transcranial magnetic stimulation, which couples high intensity magnetic fields to local electrical currents in neural systems. NPs confer to these techniques a major potential for pushing forward the attainable spatial resolution, thanks to the possibility to co-localize electromagnetic fields with the NPs, and widening the penetration depth of these techniques, thanks to a possible NPs' delivery through peripheral injection or their capability to cross the blood brain barrier.

Several approaches demonstrated to be effective in stimulating neurons via photopotential/current generation from visible or infrared light absorbed in quantum confined NPs, quantum dots (QDs), and QD films. Films of HgTe QDs in conjunction with poly(Diallyl dimethyl ammonium) chloride (PDDA) were interfaced to a model neuron cell line, which showed induced action potential firing (532 nm, 500 ms pulses @ 800 mW/cm^2^) nominally attributed to a resistive coupling of the neurons with the NPs interface (Pappas et al., 2007). Another report highlighted the modifications of neuronal membrane ionic conductance by induced electric dipole generation upon photostimulation of CdSe and CdTe QD films and micro-probes (Lugo et al., 2012).

Nanostructured PbSe films on glass microtips have also been reported to effectively depolarize neurons thanks to a NIR-induced local electric field (Zhao et al., 2009). More recently, semiconducting NRs on carbon nanotube (CNT) surfaces were exploited to obtain enhanced charge separation, and effectively stimulated action potential firing in blind chick retinas upon 405 nm illumination (Bareket et al., 2014). The latter finding not only demonstrates the potential of the platform for vision recovery, but it also represents to date the system for neural photostimulation that employs quantum confinement with the lowest stimulation threshold (3 mW/cm^2^).

The organic photovoltaic blend composed by poly(3-hexylthiophene) and phenyl-C61-butyric acid methyl ester (P3HT:PCBM) deposited on a conducting glass substrate evoked the generation of action potentials in cultured neurons upon short pulses of green light illumination (Ghezzi et al., 2011). A similar interface, composed by P3HT on conductive glass, was able instead to trigger the excitability of explanted dystrophic retinas in response to 10 ms light pulses down to 1 μW/mm^2^ (Ghezzi et al., 2013).

Recently, another application that exploits NP films as neural interface has been reported for optogenetic activation. While infrared light allows deep penetration into the brain, no specific infrared-sensitive optogenetics opsins are available. Thus, a promising strategy to circumvent this problem is the use of upconverting NPs that allow localized emission of visible light, matching opsin's peak absorption wavelength. Light pulses of 980 nm delivered to NaYF_4_:Yb^3+^/Tm^3+^ particles were sufficient to activate channelrhodopsin-2 (ChR2) and the subsequent action potential firing in hippocampal neurons grown onto the interface (Shah et al., 2015). Thanks to the tunable absorption spectrum of the NPs, the versatility of such system was reported by the activation and firing of neurons expressing other opsins, such as C1V1 or mVChR1 (Hososhima et al., 2015).

Notably, all optically driven effects involving charge separation to drive neural stimulation have thus far been carried out on NPs deposited in form of thin films. Although, very promising, further characterization and development of these nanostructures is needed to make their introduction to neural systems *in vivo* less invasive, for example by testing the possibility to inject them directly from their colloidal form while maintaining effectiveness in neural stimulation.

Magnetic nanoparticles are also emerging as neurostimulation transducers for the control of neural activity through locally generated magnetic and/or electric field. Recently, CoFe_2_O_4_-BaTiO_3_ NPs were injected into the bloodstream of mice and brought across the blood-brain barrier by exploiting the dragging force of a permanent magnet. A low energy a.c. magnetic field (100 Oe @ 0-20 Hz) was then able to modulate the brain activity as recorded by electroencephalography (Guduru et al., 2015). This novel method to wirelessly elicit neuronal activation has opened a new page in nanomedicine studies, although further investigations on the specificity and mechanisms of neural activation are needed.

## Chemical and mechanical stimulation

The use of organic and inorganic low-dimensionality systems for controlled photo- and electro-chemical release of biological agents both *in vitro* and *in vivo* has been widely reported (Gendelman et al., 2015). The main advantages offered by NPs consist in the possibility to mediate transport of chemotherapeutics across the blood-brain barrier, to combine multifunctional constructs comprising both diagnostic and therapeutic agents, and to ensure high chemical specificity and spatial resolution. A detailed review of the numerous approaches proposed in this field is well beyond the scope of this mini-review. However, it is important to mention that, in the large majority of reports, NPs acted as mere *physical carriers* of biological agents, without covering specific functional roles in the chemical interaction with cells and tissues. Nevertheless, one notable example in the direction of active chemical transducers made use of polypyrrole (ppy) NPs embedded in microscale composite hydrogels for remotely controlled release of biomolecules (Li et al., 2015). In this case, ppy-NPs acted as light-sensitive, photo-thermal transducers, which released neurotransmitters (e.g., glutamate) upon NIR excitation. While taking benefit from the nanostructured system, this approach is still hampered in chronic experiments by the impossibility to replenish the microgel within a living organism.

Finally, a huge application potential for NPs was proposed in the field of mechano-transduction, i.e., in the conversion of a mechanical stimulus into an electrochemical effect (Gillespie and Walker, 2001). The mechanism underlying this phenomenon is yet to be fully understood, but it is thought to be mediated by stretch-activated ion channels, present in all cell types and directly influenced by the presence of mechanical forces acting on the cellular membrane. Importantly, mechano-transduction is currently believed to be involved in signal transduction of neurons and astrocytes (Oliet and Bourque, 1993). Recently, a number of techniques have been reported that allow NPs-induced mechanical forces to be applied to specific receptors on the surface of cultured cells, modulating mechanosensitive ion channels activity, cytoskeletal mechanics, or growth factor release (Hughes et al., 2005; Sensenig et al., 2012; Kunze et al., 2015). Magnetic beads, in particular, were attached to integrin receptors or to specific antibodies on the surface of substrate-adherent cells. A high-gradient magnetic field was able to drag the particles in a given direction, exerting a localized deformation of the plasma membrane (Dobson, 2008). With a similar approach, ferromagnetic microparticles characterized by a fixed magnetization direction were attached to the cell surface through the use of specific ligand coatings. A weak external magnetic field resulted in a slight twisting of the particles and a subsequent torque onto the cell membrane, showing that forces within a few pN could initiate outgrowth and elongation of neurites or signaling transduction (Fass and Odde, 2003; Steketee et al., 2011). In a more recent work, cubic magnetic nanoparticles (ZnFeO) were shown to exert mechanical forces in the order of pN and to efficiently and reversibly modulate the gating of mechano-sensitive ion channels at the level of single ear hair cells in frogs, with unprecedented temporal (100 μs) and spatial resolution (Lee et al., 2014). Finally, induction of Ca^2+^ influx in cortical neurons incubated with starch- and chitosan-coated magnetic NPs have been exerted by a nanomagnetic force stimulation (Tay et al., 2016). A 20 % change in calcium influx upon stimulation and an increase in firing activity were justified by the implication of mechano-sensitive ion channels. These results highlight another promising route for remote control over neuronal activity with a wireless and non-invasive magnetic force.

## Concluding remarks

In this mini-review, we presented the state of the art neurostimulation techniques using NPs and NP-enhanced surfaces. We have described three main classes of neuron-interface interactions (see Table 1), provided that many of the proposed mechanisms need further investigations and that, in some cases, the nature of the interaction stands on multiple phenomena.

**Table 1:** **Comparison of different NPs driven neurostimulations modalities**.

**NPs-neuron interaction**	**Composition**	**Size**	**Neuronal model**	**Surface functionalization and complementary materials**	**Use**	**Genetics**	**Procedure**	**References**	**Stimulation**
Thermal	Fe_3_O_4_; MnFe_2_O_4_	22 nm; 6 nm	HEK 293 cells, rat hippocampal, and VTA neurons; HEK 293 cells, rat hippocampal and *C. elegans* neurons	PEG/PAA; streptavidin/PEG	Acute; acute (*in vitro*), 4–6 h (*in vivo*)	Yes	*in vitro, in vivo*	Chen et al., 2015; Huang et al., 2010	RF magnetic field
	AuNRs and AuNPs; AuNRs; AuNRs	20 nm (NPs); 15 × 80 nm; 18 × 71 nm	Rat auditory neurons; rat sciatic nerve; rat hippocampal neurons	Silica; NH2-PEG	15–17 h; acute; acute and up to 9 h incubation	No	*in vitro*; *in vivo*; *in vitro*	Yong et al., 2014; Eom et al., 2014; Yoo et al., 2014	NIR
	AuNPs; fluorescent dyes	20 nm; 6 nm	Rat dorsal root ganglion neurons, mouse hippocampal slices; rat primary cortical neurons	Ts1	Acute	No	*in vitro*	Carvalho-de-Souza et al., 2015; Farah et al., 2013	Optical
	BaTiO_3_	479 nm	SH-SY5Y neuroblastoma cells	Gum Arabic	24 h	No	*in vitro*	Marino et al., 2015	Ultrasound
	CoFe_2_O_4_—BaTiO_3_	30 nm	Mouse cortex	GMO	Acute	No	*in vivo*	Guduru et al., 2015	Magnetic
Electric and electromagnetic	PbSe	100 nm	Rat brain slices	–	Acute	No	*ex-vivo*	Zhao et al., 2009	NIR
	NaYF_4_:Yb^3+^/Tm^3+^; NaYF4:Sc/Yb/Er	47 nm	Mouse hippocampal neurons; ND7/23 cells	PLGA scaffold; collagen scaffold	Culture incubation time	Yes	*in vitro*	Shah et al., 2015; Hososhima et al., 2015	NIR
	CdTe and CdSe QDs; CNT + CDSe/CdS NRs; HgTe QDs	3–4 nm; 40 × 5 nm	LnCap and mouse cortical neurons; embryonic chick retina; NG108 neuroblastoma × glioma cells	Tripeptide-glutathione; PDDA scaffold and PAA	Culture incubation time; acute; culture incubation time	No	*in vitro*; *ex-vivo*;*in vitro*	Lugo et al., 2012; Bareket et al., 2014; Pappas et al., 2007	Optical
Chemical and electrochemical	ZnFeO; magnetic beads	20–120 nm; 4.5 nm	Frog inner ear hair cells; chick neurons	Silica and PEG; anti-b1 integrin antibody	Acute	No	*in vitro*	Lee et al., 2014; Fass and Odde, 2003	RF magnetic field
	Polypyrrole	10 to 20 nm	Rat hippocampal and visual cortex neurons	pNIPAM hydrogel scaffold	Acute	No	*in vitro, in vivo*	Li et al., 2015	NIR
	Fe_3_O_4_	250–2700 nm	COS-7 cells	Monoclonal anti-His antibody, Ni-NTA and RGD peptide	Acute	Yes	*in vitro*	Hughes et al., 2005	Static magnetic field

In addition to the ability of modulating neuronal activity, a key issues critical for the application of these approaches to neuro-prosthetics are the possible sources of toxicity or tissue damage. One possible way to reduce the noxious impact of NPs in neural tissues is the functionalization of the surface, a procedure that was adopted by all the reports discussed here, and demonstrated also to improve either the Z-potential, the co-localization of the NPs with the tissue, or the specificity of the functional outcome. Overheating could be a harmful general effect related to any kind of energy release in tissues and organs and may represent a potential threat for NPs neural stimulation. Another possible noxious agent is represented by the generation of chemical reactants, like oxygen species, that could interfere with the viability of cells and tissues. The incubation time and the size of the NPs play finally another important role in determining the level of damage associated with the stimulation method according to the cells internalization dynamics. Interestingly, most of the reports described here experiment indeed acute administration of NPs, leaving the question open about the efficacy upon chronic exposure.

In addition, some of the presented techniques interestingly rely on the use of optogenetics to enhance or drive the NPs-induced stimulation. The application of optogenetics to humans currently suffers of drawbacks and uncertainties regarding the safety and long-term efficacy of gene therapy. Moreover, optogenetics alone is a strong competitor for NPs driven neurostimulation in terms of specificity and spatial resolution.

It is also worth mentioning the lack for a suitable way to electrically stimulate by means of NPs, if they are not in contact with an anodic/cathodic circuit, although the possibility may exist to modulate neuronal activity by the generation of local electric fields by un-contacted NPs (Zhao et al., 2009).

Another important feature emerging from this mini-review is that most of the effective ways to trigger time-controlled and targeted changes in the electrophysiological state of neuronal membranes and their consequent action potential firing has been achieved with inorganic nanoparticles.

In conclusion, NPs represent a novel transduction platform that matches the increasing demand for spatial and targeted specificity in the treatment of neurological disorders. However, very few *in vivo* trials have been produced so far exploiting NPs as vehicle for the modulation of neural activity, demonstrating that the research in this field is still at a very early stage. Nevertheless, we believe that the striking results in neural stimulation by injection of magnetic NPs or AuNRs show the potential impact of these technologies as new therapeutic tools for neuronal injury, neuropathologies or chronic pain, just to mention a few. Next steps toward medical practice will include a more robust understanding of the mechanisms underlying the stimulation procedures and reliable chronic exposure tests.

## Author contributions

EC, MA, and PF contributed to the conception of the work and the writing of the manuscript. FB and GL revised the work critically for important intellectual content and helped correcting the final version of the manuscript. All authors read and approved the final version of the manuscript.

### Conflict of interest statement

The authors declare that the research was conducted in the absence of any commercial or financial relationships that could be construed as a potential conflict of interest. The reviewer AM declared that she is a postdoctoral fellow in the handling Editor's laboratory and the handling Editor states that the process nevertheless met the standards of a fair and objective review.
